# The zipper groups of the amyloid state of proteins

**DOI:** 10.1107/S0907444912050548

**Published:** 2013-03-09

**Authors:** James C. Stroud

**Affiliations:** a Department of Chemistry and Biochemistry, Howard Hughes Medical Institute, UCLA-DOE Institute for Genomics and Proteomics, University of California at Los Angeles, Box 951570, Los Angeles, CA 90095-1570, USA

**Keywords:** amyloid spine, steric zippers, zipper groups, symmetry, group theory, amyloid fibers

## Abstract

Fibrous proteins in the amyloid state are found both associated with numerous diseases and in the normal functions of cells. Amyloid fibers contain a repetitive spine, commonly built from a pair of β-sheets whose β-strands run perpendicular to the fiber direction and whose side chains interdigitate, much like the teeth of a zipper. In fiber spines known as homosteric zippers, identical protein segments sharing identical packing environments make the two β-sheets. In previous work based on atomic resolution crystal structures of homosteric zippers derived from a dozen proteins, the symmetries of homosteric zippers were categorized into eight classes. Here, it is shown through a formal derivation that each homosteric zipper class corresponds to a unique set of symmetry groups termed ‘zipper groups’. Furthermore, the eight previously identified classes do not account for all of the 15 possible zipper groups, which may be categorized into the complete set of ten classes. Because of their foundations in group theory, the 15 zipper groups provide a mathematically rigorous classification for homosteric zippers.

## Abstract 2

A formal derivation is provided of the 15 symmetry groups (zipper groups) available to the amyloid homosteric zipper.

## 1. Introduction   

Amyloid fibers were first found associated with denatured proteins and diseases (Eisenberg & Jucker, 2012 ▶), but have more recently been discovered in a variety of normal cellular processes (Chapman *et al.*, 2002 ▶; Si *et al.*, 2003 ▶; Fowler *et al.*, 2006 ▶; Maji *et al.*, 2009 ▶; Kato *et al.*, 2012 ▶). Apparently, evolution has harnessed this tight but reversible mode of protein association for numerous biological functions.

**Figure 1:**
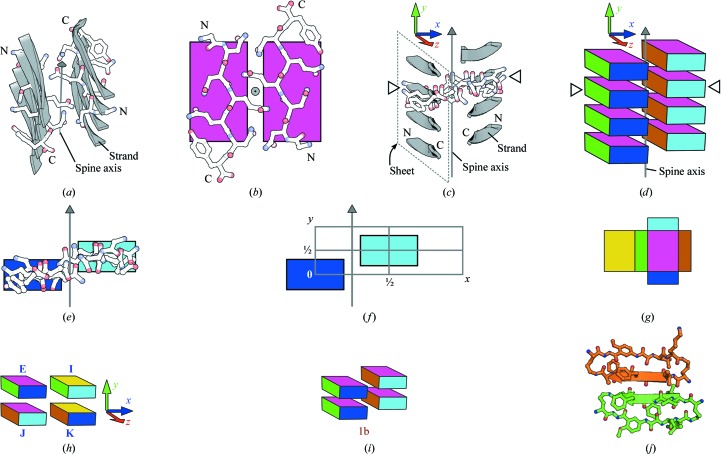
(*a*) Overview of a GNNQQNY amyloid spine (PDB entry 1yjp; Nelson *et al.*, 2005 ▶). Four β-strands from each of the two spinal β-sheets are shown. β-­Strands are depicted in gray as cartoons. One β-strand from each β-sheet is also shown as sticks with N- and C-termini labeled. All strands of a given sheet in this spine run in the same direction from the N-terminus to the C-terminus. The spine axis is shown as a gray arrow running through the center of the spine. Here, the spine axis is oblique to the viewer. (*b*) View down the GNNQQNY spine axis showing one pair of β-strands, each from a different β-­sheet. The arrow representing the spine axis in (*a*) points towards the viewer here (gray circle with dot). The N- and C- termini of the two β-strands are labeled. The magenta laminae are faces of boxes that represent the two β-strands and indicate the orientation of the β-strands, as described for (*d*). Here, the magenta faces are orthogonal to the spine axis. (*c*) A view of the GNNQQNY spine looking perpendicular to the spine axis. This vantage point is similar to that in (*e*), but slightly rotated around the spine axis. The *x*, *y* and *z* axes of the coordinate system for this GNNQQNY spine are shown in blue, green and red, respectively. The *y* axis is parallel to the spine axis. One β-sheet of the spine is outlined by a dashed parallelogram. One β-strand from each β-sheet is labeled at the N- and C-termini. Open triangles indicate two β-strands that are also indicated in (*d*). (*d*) The GNNQQNY spine is represented as stacks of boxes with colored faces, seen from the same vantage point as in (*c*). Each box represents a different β-strand. Colors indicate orientation such that various landmarks of the β-strands point towards the different colored faces of a representative box. For this GNNQQNY example, landmark features and the faces to which they point are as follows: N-terminus of each β-strand, cyan face; C-terminus, blue face; side chain of the first Q, amber face; backbone carbonyl of the first Q, magenta face; side chain of the second Q, green face; backbone carbonyl of the second Q, yellow face. (*e*) A view down the *z* axis of the GNNQQNY spine, showing only two β-strands, one from each sheet. The two β-strands are depicted as both sticks and colored boxes. Here, the blue and cyan faces of the boxes are in the *xy* plane, which is also the plane of the figure, so the other faces of the boxes are not visible. The spine axis is shown as a gray arrow. This vantage point, similar to those in (*c*) and (*d*), is created from the vantage point of (*b*) by a 90° rotation around the *x* axis, where the now hidden magenta faces point towards the top of the figure. (*f*) The homosteric zipper lattice (gray grid) is made from half-unit translations along the *x* and *y* axes at *z* = 0. The grey arrow represents the spine axis, which is defined to be at *x* = ¼ and *z* = 0. (*g*, *h*, *i*) The rotations **E**_3_ (identity), **I**_3_ (180° rotation around *x*), **J**_3_ (180° around *y*) and **K**_3_ (180° around *z*) on a single β-strand are illustrated using the colored box. This box is set into the zipper lattice to schematize homosteric zippers, as in (*d*) and (*i*) and in Fig. 2 ▶. β-Strands run from the N-terminus to the C-terminus parallel to the *z* axis (from the cyan side of the box to the blue side). The *y* axis is perpendicular to the magenta and yellow sides. The *x* axis, which is perpendicular to the green and amber sides, runs through the interface of the two spinal β-sheets. (*g*) The exterior surface of the box is shown as if the box were unfolded. (*h*) The box is shown in four orientations representing the four rotations on the reference (labeled with the identity operation, **E**). The subscripts have been dropped from the rotation labels **E**, **I**, **J** and **K**. (*i*) The GNNQQNY homosteric zipper (zipper group 1b) is depicted as two stacks of colored boxes. Two repeats (unit cells) are shown. The two β-sheets are staggered along the *y* axis as dictated by the zipper group 1b generators (**E** and **Jxy**), producing 2_1_ symmetry along the spine axis. (*j*) The atomic resolution crystal structure of mcLVFFA (Liu *et al.*, 2011 ▶) contains a parallel, face=back β-sheet. The structure of one dimer of a macrocycle tetramer is shown as a ball-and-stick model with the β-strands (LVFFA) represented as cartoons. In the top macrocycle (amber), the residues Leu1, Phe3 and Ala5 project toward the viewer, given the numbering 1–5. In the bottom macrocycle (green), the residues Val2 and Phe4 project toward the viewer.

Although models of amyloid fibers are varied (Nelson & Eisenberg, 2006 ▶), several models contain a structural feature known as the amyloid spine (Fig. 1 ▶ *a*). The spine consists of a pair of β-sheets that run the length of the fiber (Nelson *et al.*, 2005 ▶). The β-strands of the two spinal β-sheets are small adhesive segments of potentially larger polypeptide chains that constitute the fiber (Sambashivan *et al.*, 2005 ▶). β-­Hydrogen bonding within the spinal β-sheets mediates β-­strand adhesion along the spine axis and provides cooperative forces that bestow fibers with high thermodynamic stability (Nelson *et al.*, 2005 ▶; Tsemekhman *et al.*, 2007 ▶). Tightly interdigitated side chains of the β-strands (Fig. 1 ▶ *b*) bind the two β-sheets together in a spine geometry termed a ‘steric zipper’ (Nelson *et al.*, 2005 ▶; Sawaya *et al.*, 2007 ▶). Along with numerous examples from single-crystal diffraction (Eisenberg & Jucker, 2012 ▶), the steric zipper model is consistent with models derived from other types of data. For instance, a fiber-diffraction model of polyglutamine satisfies the requirements for a steric zipper (Sikorski & Atkins, 2005 ▶), as do models of amyloid-β fibrillar oligomers derived from powder diffraction (Stroud *et al.*, 2012 ▶).

A variety of steric zipper symmetries have emerged from X-­ray crystallographic studies of the adhesive segments of amyloid fibers (Nelson *et al.*, 2005 ▶; Sawaya *et al.*, 2007 ▶; Wiltzius *et al.*, 2009 ▶; Colletier *et al.*, 2011 ▶). In these studies the spinal β-­sheets have identical β-strands running nearly perpen­dicular to the spine axis (Fig. 1 ▶ *c*). Additionally, all of the β-­strands in a given spine have identical packing environments. Given the growing catalog of amyloid interactions, it is useful to enumerate the symmetries of these interactions, just as it was useful to enumerate the space groups: all possible ways to pack identical objects. Here, I derive and illustrate all possible steric zipper interactions between identical protein segments wherein all segments share identical packing environ­ments: the so-called homosteric zippers.

In all steric zipper X-ray structures determined to date, two β-sheets form the spine (Sawaya *et al.*, 2007 ▶). The spinal β-­sheets of a zipper are identical in that (i) both β-sheets are parallel (*i.e.* all β-strands run in the same direction) or antiparallel (alternate β-strands run in opposite directions) and (ii) both β-sheets have the same β-hydrogen-bonding pattern such that identical residues make the same β-hydrogen-bonding contacts in both β-sheets. Additionally, these atomic resolution structures reveal steric zippers that consist exclusively of β-strands of a single sequence, called ‘homosteric zippers’ (Eisenberg & Jucker, 2012 ▶). Homosteric zippers differ from ‘heterosteric zippers’, in which the β-strands of the spine have more than one sequence.

Sawaya and coworkers classified the known homosteric zipper structures using three class constraints to specify zipper features: (i) how each β-strand interacts with its nearest-neighboring β-strands in the same β-sheet (‘parallel’ or ‘antiparallel’), (ii) whether the same sides of both β-sheets are up (‘up–up’) or whether one is up and other is down (‘up–down’) or whether both can be rotated by 180° around an axis perpendicular to the β-sheets to yield identical β-sheets (‘up=down’) and (iii) whether identical (‘face-to-face’) or opposite (‘face-to-back’) faces of the β-­sheets create the zipper interface or whether the β-sheets can both be flipped around an axis parallel to the spine axis to yield the same β-­sheets (‘face=back’). Eight classes of homo­steric zippers arise from the possible combinations of these class constraints (Sawaya *et al.*, 2007 ▶).

The group-theoretic treatment herein establishes a mathematically rigorous classification of 15 zipper groups and shows that the full set of homosteric zipper classes should expand to ten. To enumerate all homosteric zipper classes, I describe their symmetries in terms of a coordinate system in which the homosteric zipper spine is oriented with its β-­hydrogen bonds (backbone C=O and N—H groups) running nearly parallel to the *y* axis and with its β-­strands running nearly parallel to the *z* axis (Fig. 1 ▶ *c*). The spine axis is parallel to the *y* axis, lying in the interface between the β-sheets at a defined location in the coordinate system (Fig. 1 ▶ *f*).

**Figure 2:**
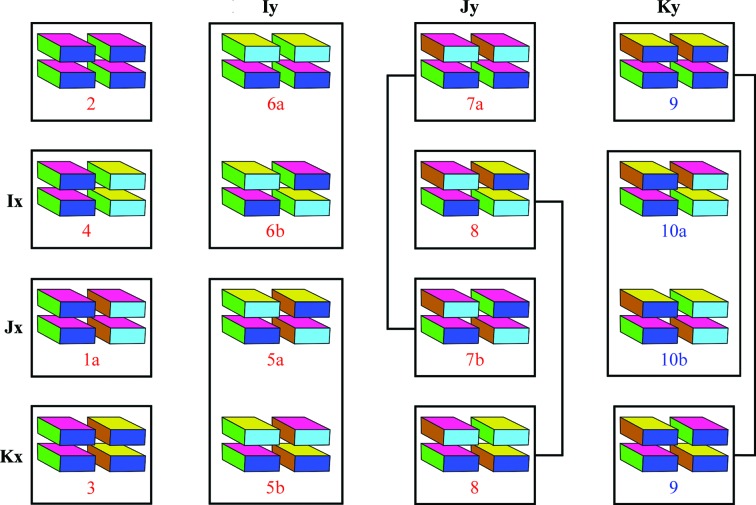
Relationship of zipper groups to the ten homosteric zipper classes, eight of which have previously been described (Sawaya *et al.*, 2007 ▶). Zipper groups are organized by the rotation and translation components of the generators. Zipper groups that satisfy previously identified homosteric zipper classes are labeled in red. Zipper groups that do not satisfy any of the previously identified classes are labeled in blue. **E** (identity) and the generators in the row and column headings produce the zipper groups listed in each cell of the chart (see also Table 2 ▶). Not shown here is zipper group 1b (shown in Fig. 1 ▶ *i*), which is generated by **E** and **Jxy**. Zipper groups that satisfy the same homosteric zipper classes are shown connected by a line or boxed together.

By enumerating all possible combinations of symmetry operations (Table 1 ▶), I demonstrate the existence of 15 distinct symmetry groups, termed ‘zipper groups’ (Table 2 ▶). Each of the eight homosteric zipper classes identified by Sawaya and coworkers corresponds to a subset of the zipper groups, accounting for 12 of the 15 zipper groups. Two novel classes of homosteric zippers correspond to the three remaining zipper groups (Fig. 2 ▶), extending the number of homosteric zipper classes to ten.

## 2. Methods: derivation of the 15 homosteric zipper groups   

### 2.1. The zipper-group positions   

I represent the homosteric zipper lattice by a set of translations, {**e**_3_, **x**_3_, **y**_3_, **xy**_3_} (1), such that one β-sheet is centered on **e**_3_ and another on **x**_3_, with the spine axis halfway between **e**_3_ and **x**_3_ and running parallel to the *y* axis (Fig. 1 ▶ *f*), 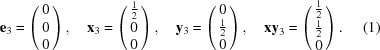
β-Strands that occupy a zipper lattice can be in one of four orientations (2) that correspond to a reference (**E**_3_) or π rotations (**I**_3_, **J**_3_ and **K**_3_) around each of the three principal axes (*x*, *y* and *z*, respectively), 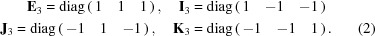
A multicolored box (shown as if unfolded in Fig. 1 ▶ *g*) illustrates these orientations. Different sides of the box are visible depending on the orientation (Fig. 1 ▶ *h*).

Combining the translations {**e**_3_, **x**_3_, **y**_3_, **xy**_3_} with rotations {**E**_3_, **I**_3_, **J**_3_ and **K**_3_} produces positions. For example, **J**_3_ and **x**_3_ combine to produce the position **Jx**. Positions are represented as 4 × 4 projective transformation matrices. An example is shown in (3):
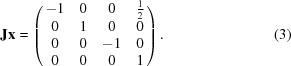
The resulting positions (**E**, **I**, **J**, **K**, **x**, **Ix**, **Jx**, …, **Kxy**) form a finite group under matrix multiplication wherein the translation component (elements [1 4], [2 4] and [3 4]) of the product matrix is under modulo 1. This modulo operation simply implies that a translation as large or larger than a unit cell moves to a position in a different unit cell.

### 2.2. Ten generators produce the 15 zipper groups   

Every symmetry group available to zipper lattices may be produced from ten generators (Table 1 ▶) combined such that no more than one generator with a given translation (**e**_3_, **x**_3_, **y**_3_ or **xy**_3_) is used in each combination. This requirement simply implies that no two segments may occupy the same lattice position. The resulting 15 distinct zipper groups are layer groups of non-enantiomorphic objects expanded to include multiple settings for several of the layer groups (Table 2 ▶).

To illustrate a zipper group, I place multicolored boxes that represent specific orientations (Fig. 1 ▶ *h*) into a lattice. An example is Fig. 1 ▶(*i*), which illustrates two repeats of a GNNQQNY homosteric zipper (PDB entry 1yjp; Nelson *et al.*, 2005 ▶) which belongs to zipper group 1b. Fig. 1(*f*) depicts one unit cell of the zipper group 1b lattice.

### 2.3. Zipper groups have layer-group symmetry   

Although the zipper-group settings are different from the standard layer-group settings, the zipper groups have layer-group symmetry. For example, the generators for zipper group 1b (**E** and **Jx**) combine to generate layer group 8 with *C*_2_^*y*^ point symmetry. This symmetry is demonstrated by changing the basis such that the translation components of the two positions shift by (−¼ 0 0)*^T^* (4),
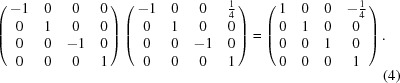
Different zipper groups may have the same layer-group symmetry (Table 2 ▶). For example, zipper groups 1b and 7a both have layer group 9 symmetry. These two groups are distinguished by their settings, which places symmetry axes at different locations in the two zipper groups. Zipper group 1b has 2_1_ symmetry along the spine axis at *x* = ¼. In contrast, zipper group 7a has 2_1_ symmetries coincident with the centers of the β-sheets at *x* = 0 and *x* = ½. Because of the stereochemistry of β-hydrogen bonding, some symmetries along the *z* axis in zipper groups 9, 10a and 10b are imperfect.

## 3. Discussion   

### 3.1. Nomenclature of the zipper groups reflects their relation to the homosteric zipper classes   

Because zipper-group symmetries satisfy homosteric zipper classes, the names of the zipper groups have been assigned to reflect the names of homosteric zipper classes (Table 2 ▶). For example, zipper groups 1a and 1b satisfy the homosteric zipper class 1 constraints (parallel, up–up, face=back). Fig. 2 ▶ illustrates the relationships between zipper groups, their generators and the homosteric zipper classes.

### 3.2. Zipper groups predict novel homosteric zipper symmetries   

A complete enumeration of zipper groups (Table 2 ▶) reveals that three zipper groups (9, 10a and 10b) remain after accounting for all eight previously identified homosteric zipper classes (Sawaya *et al.*, 2007 ▶). Zipper groups 9, 10a and 10b satisfy the novel combination of parallel, face=back. Zipper group 9 differs from 10a and 10b in that both β-sheets of zipper group 9 run in the same direction along *z* (a class constraint termed ‘head-to-head’; Fig. 2 ▶). Zipper groups 10a and 10b have β-sheets that run in opposite directions along *z* (‘head-to-tail’). Although not yet observed in atomic structures of homosteric zippers, a parallel, face=back β-sheet has been observed in the atomic resolution crystal structure of the mcLVFFA macrocyclic β-sheet mimic (Liu *et al.*, 2011 ▶). In this structure (Fig. 1 ▶ *j*), the sequence LVFFA makes a parallel, face=back β-sheet, suggesting that LVFFA has the potential to form zipper group 9, 10a or 10b.

### 3.3. Rationale for the preponderance of certain zipper groups   

Some zipper groups are observed in crystal structures more frequently than expected, perhaps because symmetry influences the stability of the amyloid spine. For example, the symmetries of several zipper groups require that interacting β-­strands from different β-sheets be in the same plane, where the plane is perpendicular to the spine axis. Such zipper groups are described as ‘eclipsed’ (Table 2 ▶). Of the 15 zipper groups, seven are eclipsed. Yet among all 44 published crystallo­graphic homosteric zippers (Table 3 ▶), eclipsed zipper groups are rare, comprising only seven of the 44 zippers. This bias towards ‘staggered’ zipper groups (groups that are not eclipsed) may arise from the profile method used to identify amyloidogenic segments, which employs the zipper group 1b NNQQNY zipper as a template (Nelson *et al.*, 2005 ▶; Thompson *et al.*, 2006 ▶). However, a strong bias towards staggered zippers exists even when group 1b zippers are excluded, with only seven of the 24 remaining zippers being eclipsed. The bias towards staggered zippers may reflect the fact that staggered zippers more readily interdigitate than eclipsed zippers, increasing the surface complementarity and hence the energetic favorability of sheet adhesion.

### 3.4. Zipper-group pseudo-symmetry is observed in some crystal structures   

In experimental structures, the strict layer-group symmetries of several zipper groups are broken by a shift along the *z* axis (termed ‘*z*-shift’) of one β-sheet relative to the other (Table 2 ▶, ‘Alternate Symmetry’). This *z*-shift produces a 2_1_ screw from *C*_2_^*z*^ point symmetry of zipper groups 3, 8 and 9. Similarly, the *z*-shift creates 2_1_ symmetry from *C*_2_^*x*^ point symmetry of zipper groups 6a and 6b. The potential for some of these 2_1_ screw axes in *x* and *z* has been recognized previously (Sawaya *et al.*, 2007 ▶). In zipper groups 5a and 5b, the *z*-shift completely removes any symmetries in *x* and *z*. Because zipper groups are defined by generators with only *x* and *y* translations, the *z*-shift does not influence the ability of the affected zipper groups to satisfy homosteric zipper classes.

## 4. Conclusion   

Here, I develop a mathematically rigorous classification of homosteric zippers using group theory to derive the 15 zipper groups that specify all possible symmetries available to homosteric zippers. Zipper groups extend previous work in which eight symmetry classes of homosteric zipper spines were identified from crystal structures and from an intuitive analysis of the ways that pairs of β-sheets can interact (Sawaya *et al.*, 2007 ▶). Zipper groups may be categorized such that the complete homosteric zipper classification developed by Sawaya and coworkers expands to ten classes. Subsequent to the work of Sawaya and coworkers, a sheet satisfying this expanded set of symmetries was observed in a crystal structure (Liu *et al.*, 2011 ▶). I anticipate that structures of amyloid spines belonging to the new homosteric zipper symmetries will be discovered in the future.

## Figures and Tables

**Table 1:** Representations of the zipper-group generators Indices listed in the first column specify elements that differ from the **1**_4_ identity projective transformation matrix.

Element	**E**	**Ix**	**Jx**	**Kx**	**Iy**	**Jy**	**Ky**	**Ixy**	**Jxy**	**Kxy**
1 1	1	1	1	1	1	1	1	1	1	1
2 2	1	1	1	1	1	1	1	1	1	1
3 3	1	1	1	1	1	1	1	1	1	1
1 4	0				0	0	0			
2 4	0	0	0	0						

**Table 2:** The 19 possible combinations of generators produce 15 distinct zipper groups Zipper-group names are based on the homosteric zipper classes described previously (Sawaya *et al.*, 2007 ▶). Underlined positions are group generators. Each homosteric zipper class corresponds to two distinct zipper groups except for homosteric zipper classes 2, 3, 4, 8 and 9. The penultimate column indicates alternate symmetry that arises from the shift of one -sheet relative to the other along the *z* axis (described in the text). The last column indicates whether the symmetry of the zipper group requires the -sheets to be eclipsed, where neighboring -strands in the two -sheets are in the same plane.

Zipper group	Positions	Layer group	Principal axis	Alternate symmetry	Eclipsed
2	**E**	1 (*p*111)			N
4	**E**, **Ix**	9 (*p*2_1_11)	*x* (2_1_)		N
6a	**E**, **Iy**	8 (*p*211)	*x* (twofold)	*p*2_1_11	Y
4	**E**, **Ixy**	9 (*p*2_1_11)	*x* (2_1_)		N
1a	**E**, **Jx**	8 (*p*121)	*y* (twofold)		Y
7a	**E**, **Jy**	9 (*p*12_1_1)	*y* (2_1_)		N
1b	**E**, **Jxy**	9 (*p*12_1_1)	*y* (2_1_)		N
3	**E**, **Kx**	3 (*p*112)	*z* (twofold)	*p*112_1_	N
9	**E**, **Ky**	3 (*p*112)	*z* (twofold)	*c*112	N
3	**E**, **Kxy**	3 (*p*112)	*z* (twofold)	*p*112_1_	N
6_b_	**E**, **Ix**, **Iy**,** xy**	10 (*c*211)	*x* (2_1_ and twofold)	*p*2_1_11	Y
8	**E**, **Ix**, **Jy**, **Kxy**	21 (*p*2_1_2_1_2)		*p*2_1_2_1_2_1_	N
10a	**E**, **Ix**, **Ky**, **Jxy**	21 (*p*2_1_2_1_2)			N
5a	**E**, **Jx**, **Iy**, **Kxy**	19 (*p*222)		*p*121	Y
7b	**E**, **Jx**, **Jy**, **xy**	10 (*c*121)	*y* (2_1_ and twofold)		Y
10b	**E**, **Jx**, **Ky**, **Ixy**	20 (*p*2_1_22)			Y
5b	**E**, **Kx**, **Iy**, **Jxy**	20 (*p*22_1_2)		*p*12_1_1	Y
8	**E**, **Kx**, **Jy**, **Ixy**	21 (*p*2_1_2_1_2)		*p*2_1_2_1_2_1_	N
9	**E**, **Kx**, **Ky**, **xy**	3 (*b*112)	*z* (twofold)	*i*112	N

**Table 3:** The zipper groups of 44 published homosteric zipper crystal structures Zipper-group symmetries are related to crystallographic symmetry. The layer-group symmetries of the zippers are shown in the column ‘Layer symmetry’. The ‘Cryst’ column shows the crystallographic symmetry of the structure. The following annotations are used. , : for a structure, the crystallographic symmetry bearing the given annotation produces the zipper symmetry bearing the same annotation. : noncrystallographic symmetry or pseudosymmetry. a: the twofold component of the *C*2 crystallographic symmetry creates the twofold component of the *C*2 zipper symmetry. b: the 2_1_ crystallographic symmetry creates the 2_1_ component of the *C*2 zipper symmetry, c: the same crystal structure has two different zipper interfaces. Parentheses: the strict layer-group symmetry indicated is broken by a *z*-shift.

	Sequence	PDB code	Layer symmetry	Layer group	Zipper group	Cryst
1	GDVIEV	3sgs	*p* 2_1_11	9	4	*P*12_1_1
2	AIIGLM	2y3j	*p*111	1	2	*P*111
3	MVGGVVIA	2y3k	*p*111	1	2	*P*111
4	MVGGVVIA	2y3l	*p*12_1_1	9	7a	*P*12_1_1
5	KLVFFA	2y29	*p*12_1_1	9	7a	*P*2_1_ 2_1_2_1_
6	KLVFFA	2y2a	*p*12_1_1	9	7a	*P* 2_1_2_1_2_1_
7	GAIIGL	3pzz	*p* 211	10	6b	*P*111
8	NKGAII	3q2x	*p*12_1_1	9	1b	*P*12_1_1
9	KLVFFA	3ow9	*c*1^a^21	10	7b	*C*1^a^21
10	VQIVYK	3ovl	*p*12_1_1	9	1b	*C*121
11	GGVLVN	3ppd	*p*12_1_1	9	1b	*P*2_1_2_1_ 2_1_
12	MMHFGN	3nve	*p* 211	8	6a	*P*12_1_1
13	IIHFGS	3nvf	*p*121	8	1a	*P*2_1_2_1_ 2
14	MIHFGN	3nvg	*p*12_1_1	9	1b	*P*2_1_ 2_1_2_1_
15	MIHFGND	3nvh	*p*12_1_1	9	1b	*P*2_1_ 2_1_2_1_
16	LSFSKD	3loz	*p*12_1_1	(20)	5b	*P*12_1_1
17	GYMLGS	3nhc	*p* 2_1_ 2_1_ 2_1_	(21)	8	*P* 2_1_ 2_1_2_1_
18	GYVLGS	3nhd	*p* 2_1_ 2_1_ 2_1_	(21)	8	*P* 2_1_ 2_1_2
19	LVEALYL	3hyd	*p*12_1_1	9	1b	*C*121
20	HSSNNF	3fpo	*p* 2_1_11	9	4	*P*12_1_1
21	VQIVYK	3fpq	*p*12_1_1	9	1b	*C*121
22	NFLVHS	3fr1	*p*12_1_1	9	7a	*P*2_1_ 2_1_2_1_
23	NFLVHSS	3fth	*c* ^b^211	10	6b	*P*1^b^2_1_1
24	NVGSNTY	3ftk	*p*12_1_1	9	1b	*P*12_1_1
25	NVGSNTY	3ftl	*p*12_1_1	9	1b	*P*12_1_1
26	SSTNVG	3ftr	*p*12_1_1	9	1b	*P*2_1_ 2_1_2_1_
27	NNQNTF	^c^ 3vfa	*p*12_1_1	9	1b	*P*12_1_1
28	NNQNTF	^c^ 3vfa	*p*12_1_1	9	1b	*P*12_1_1
29	AILSST	3fod	*p* 2_1_ 2_1_ 2	21	8	*P*12_1_1
30	SSTNVG	3dg1	*p*12_1_1	9	1b	*C*121
31	NNFGAIL	3dgj	*p*12_1_1	9	1b	*P*2_1_ 2_1_2_1_
32	GGVVIA	2onv	*p* 2_1_11	9	4	*P* 2_1_2_1_2
33	SSTSSA	2onw	*p*12_1_1	9	1b	*P*2_1_ 2_1_2_1_
34	NNQQ	2onx	*p* 2_1_11	9	4	*P*12_1_1
35	MVGGVV	2okz	*p* 2_1_ 2_1_ 2_1_	(21)	8	*P*12_1_1
36	SNQNNF	2ol9	*p*111	1	2	*P*111
37	NNQQ	2olx	*p*12_1_1	9	1b	*P*2_1_ 2_1_2_1_
38	GNNQQNY	2omm	*p*12_1_1	9	1b	*P*2_1_ 2_1_2_1_
39	LYQLEN	2omp	*p*12_1_1	9	7a	*P*12_1_1
40	VEALYL	2omq	*p*12_1_1	9	7a	*P*111
41	VQIVYK	2on9	*p*12_1_1	9	1b	*P*12_1_1
42	MVGGVV	2ona	*p* 2_1_ 2_1_ 2_1_	(21)	8	*P*111
43	NNQQNY	1yjo	*p*12_1_1	9	1b	*P*12_1_1
44	GNNQQNY	1yjp	*p*12_1_1	9	1b	*P*12_1_1
